# Participant self-collected versus research coordinator-collected nasal swabs for respiratory viral surveillance following influenza-like illness

**DOI:** 10.3389/fpubh.2026.1846269

**Published:** 2026-08-19

**Authors:** Michelle Kautz, Kat Schmidt, Stephanie A. Richard, Rhonda E. Colombo, Nusrat J. Epsi, Anuradha Ganesan, Ryan C. Maves, Katrin Mende, Christina Schofield, Jay R. Montgomery, Simon Pollett, Derek T. Larson, Anthony C. Fries, Timothy H. Burgess, Tahaniyat Lalani

**Affiliations:** 1 Navy Medicine Readiness and Training Command San Diego, San Diego, CA, United States; 2 Infectious Disease Clinical Research Program, Department of Preventive Medicine and Biostatistics, Uniformed Services University of the Health Sciences, Bethesda, MD, United States; 3 The Henry M. Jackson Foundation for the Advancement of Military Medicine, Inc., Bethesda, MD, United States; 4 Madigan Army Medical Center, Tacoma, WA, United States; 5 Walter Reed National Military Medical Center, Bethesda, MD, United States; 6 Wake Forest University School of Medicine, Winston-Salem, NC, United States; 7 Brooke Army Medical Center, JBSA Fort Sam Houston, San Antonio, TX, United States; 8 The United States Airforce School of Aerospace Medicine, Dayton, OH, United States

**Keywords:** health-care worker collection, influenza-like illness, nasal swabs, respiratory viral, self-collection, surveillance

## Abstract

**Background:**

Unsupervised, self-collected nasal swabs (SCNS) are a convenient alternative to collection by health-care workers for influenza-like illness (ILI) surveillance. We compared viral respiratory pathogen detection in paired SCNS versus research coordinator-collected nasal swabs (CCNS).

**Methods:**

Adult Military Health System beneficiaries were enrolled in a prospective influenza vaccine effectiveness trial. Following vaccination, participants were contacted weekly to ascertain ILI symptoms. In the event of an ILI, participants completed a symptom diary, a SCNS, and were offered a visit for CCNS. We evaluated respiratory pathogen detection by PCR, concordance between paired swabs (Cohen’s Kappa statistic), and the impact of the timing (days post symptom onset [DPSO]) of collection on detection.

**Results:**

3,357 ILIs were reported during the study period and paired (SCNS and CCNS) swabs were obtained during 1,048 ILIs. Among the paired swabs, SCNS were generally collected earlier than CCNS (median DPSO: 3.0 versus CCNS: 7.0; *p* < 0.001) and a higher proportion reported moderate or severe symptoms at the time of SCNS collection (43.9% versus 33.3%; *p* < 0.001). Among 988 swab pairs collected 0–7 days apart, viral pathogen detection was higher in SCNS (39%) versus CCNS (31%) (*p* < 0.001; K = 0.59). Paired swabs collected within 7 DPSO (K: 0.63 versus 0.42) were associated with a higher concordance.

**Conclusion:**

SCNS are a feasible and useful alternative to CCNS for respiratory virus surveillance and research as it allows an average earlier collection time. Detection of viral pathogens is increased by obtaining swabs within 7 days of symptom onset.

**Clinical trial registration:**

https://clinicaltrials.gov/study/NCT03734237?term=NCT03734237&rank=1, NCT03734237.

## Introduction

Laboratory and survey-based surveillance efforts are essential for monitoring the temporal and geographic circulation of respiratory viruses, estimating the disease burden, and evaluating the effectiveness of vaccination strategies (1, 2). Surveillance networks have recognized the advantages of supplementing passive surveillance data from clinical and public health laboratories with cohorts of online volunteers who provide regular symptom data and self-collected nasal swabs (SCNS) for viral detection by PCR (3–5). Such decentralized enrollment strategies can improve the accuracy of incidence estimates for infections that do not result in healthcare utilization, while increasing the convenience of study participation and reducing the staffing and personal protective equipment costs. This approach gained traction during the COVID-19 pandemic, and detection rates for SCNS were found to be comparable to health care worker (HCW) collected swabs for SARS-CoV-2 and influenza in a few studies. However, most of these studies had a cross-sectional study design (i.e., both swabs performed at a clinic visit), were limited to a single viral pathogen, or were only conducted in pediatric populations (Table 1) (6–12). There are limited data from longitudinal adult cohort studies on the timing of unsupervised SCNS and clinical research coordinator collected nasal swabs (CCNS) in relation to symptom onset and its impact on viral detection.

**Table 1:** Summary of prior studies comparing self-collected nasal swabs versus healthcare worker-collected swabs in adults for detection of viral respiratory pathogens.

Reference	Study design	No. of swab pairs	Collection site	Median time from symptom onset to swab collection		Detection rate		
				HCW collect	Self collect	HCW collect	Self collect	Kappa coefficient (95% CI)
Influenza								
Dhiman (6)	Cross-sectional (ILI visit)	58^✝^	Mid-turbinate	Not reported	Not reported	33%	31%	0.88 (0.75–1.00)
Goyal (7)	Cross-sectional (ILI visit)	127	Nasal	4 days	4 days	6%	6%	0.87 (0.68–1.00)
Thind (8)	Cross-sectional sub-study (home visit for ILI)	154	Nasal	Not reported	Not reported	14%	12%	0.71 (0.55–0.88)
SARS-CoV-2								
McCulloch (9)	Cross-sectional study (CLI visit)	185	HCW: NP SC: mid-nasal	2 days	2 days	20%	17%	0.81 (0.70–0.92)
Alemany (10)	Cross-sectional (CLI visit)	130	HCW: NP SC: mid-nasal	4 days	4 days	92%	92%	0.89 (0.74–1.00)
Hanson (11)	Cross-sectional (CLI visit)	354	HCW: NP SC: nasal swab	Not reported	Not reported	23%	20%	0.90 (0.84–0.96)
Tu (12)	Cross-sectional (CLI visit)	498	HCW: NP SC: nasal	7 days	7 days	10%	10%	0.96 (0.91–0.99)
Rhino-enterovirus								
Thind (8)	Cross-sectional sub-study (home visit for ILI)	154	Nasal	Not reported	Not reported	14%	16%	0.61^ǂ^ (0.43-0.79)
Seasonal coronavirus								
Thind (8)	Cross-sectional sub-study (home visit for ILI)	154	Nasal	Not reported	Not reported	18%	16%	0.77^ǂ^ (0.63-0.90)

^✝^72 paired specimens but 25 were excluded because subjects had prior healthcare training. Results for the remaining 58 are shown. ILI: influenza-like-illness visit; CLI: COVID (Coronavirus disease) like illness.

^ǂ^This study included adults and children; kappa co-efficient for rhino-enterovirus and seasonal coronavirus detection in paired swabs among adult subjects could not be calculated as necessary data was not presented in manuscript.

CI, confidence interval; HCW, health-care worker; ILI, influenza-like illness; NP, nasopharyngeal; SC, self-collect.

We performed a sub-analysis of data from an influenza vaccine effectiveness clinical trial in adult Department of War (DoW) healthcare beneficiaries (13) to compare the detection of respiratory viruses in SCNS and CCNS, and to identify other factors associated with improved detection.

## Methods

The Pragmatic Assessment of Influenza Vaccine Effectiveness in the DoD (PAIVED) study is a randomized trial comparing the immunogenicity and protective efficacy of different licensed influenza vaccines (13). Participants were at least 18 years of age and were eligible for healthcare in a military treatment facility. Every week during influenza season, participants were asked about influenza-like illness (ILI) symptoms (fever, sore throat, and/or cough) via text or email, and participants could self-report ILI symptoms between weekly messages. A participant was considered to have an ILI if they reported cough or sore throat and at least one of the following symptom categories: (1) feverish/chills, and/or (2) muscle/body aches or fatigue, occurring ≥14 days after vaccination. Participants were asked to complete the FLU-PRO instrument (14), a validated patient-reported outcomes survey completed for seven consecutive days from ILI notification (15, 16).

The study was conducted over four influenza seasons (2018/2019, 2019/2020, 2020/2021, and 2021/2022). The SCNS and CCNS sample collection protocol varied across seasons. For this sub-analysis, we included subjects enrolled in the 2019/2020 and 2021/2022 influenza seasons, when paired SCNS and CCNS were obtained. During both seasons, participants were given a swab collection kit (VTM [MicroTest™ M4RT™ Transport Medium, Remel]) upon study enrollment, educated on how to perform a SCNS as soon as they developed ILI symptoms and were instructed to mail the swab to the study site post-collection. Participants were reminded about SCNS collection when they reported an ILI. Participants stored viral transport media containing the swabs at room temperature and mailed them to the study site using standard packaging; samples were stored at ambient temperature during transport. In-person study visits for CCNS, ideally conducted within seven days of ILI notification, were required during the 2019/2020 season until the institution of COVID-19 pandemic restrictions were implemented; thereafter, in-person visits became optional and remained optional for the 2021/2022 season.

Participants could enroll in more than one season, and nasal swabs were collected on the first three reported ILIs within a given season. SCNS and CCNS were performed by inserting a sterile polyester flocked swab in each nostril, rotating three or more times, and placing the swab in the viral transport media tube of the collection kit after labeling it with the date of collection. Additional collection kits were mailed to participants as needed.

Swabs were stored at −80 °C at the study sites and shipped weekly on dry ice to a central laboratory for testing with the NxTAG® Respiratory Pathogen Panel for the Luminex MAGPIX instrument to identify the following viruses: adenovirus; human seasonal coronavirus (hCoV) 229E, OC43, NL63, or HKU1; human bocavirus (hBCV); human metapneumovirus (hMPV); influenza (A, A/H1N1, A/H3N2, or B); human parainfluenza virus (hPIV) 1, 2, 3, or 4; respiratory syncytial virus (RSV) A or B; human rhinovirus/enterovirus (hRV/EV). A real-time PCR was performed for detection of SARS-CoV-2. Subjects with paired swabs from more than one ILI were included in this sub-analysis; each ILI contributed only one pair of swabs. We excluded swabs without collection dates (*n* = 2) or testing results (*n* = 36).

We determined the proportion of ILIs with SCNS and/or CCNS collected during each of the two seasons. Demographic data were collected at enrollment and symptom severity was collected from the FLU-PRO instrument. For paired SCNS/CCNS, we evaluated the time between symptom onset and swab collection, symptom severity at swab collection, and pathogen detection. To minimize variability in pathogen detection due to differences in collection timing between swabs, paired swabs collected between days 0–7 were utilized in the analyses. The agreement between paired SCNS and CCNS collected ≤ 7 days apart for a given ILI pathogen was evaluated using Cohen’s Kappa coefficient (K). A Kappa coefficient of 0.01–0.20 was interpreted as none to slight agreement, 0.21–0.40 as fair, 0.41–0.60 as moderate, 0.61–0.80 as substantial, and 0.81–1.00 as almost perfect agreement. We performed an additional sensitivity analysis of agreement between PCR results of paired swabs that were both collected within seven days post symptom onset (DPSO) versus when at least one swab was collected > 7 DPSO. Analyses were performed using R Statistical Software (v4.2.3; R Core Team 2023) (17).

## Results

**Figure 1:**
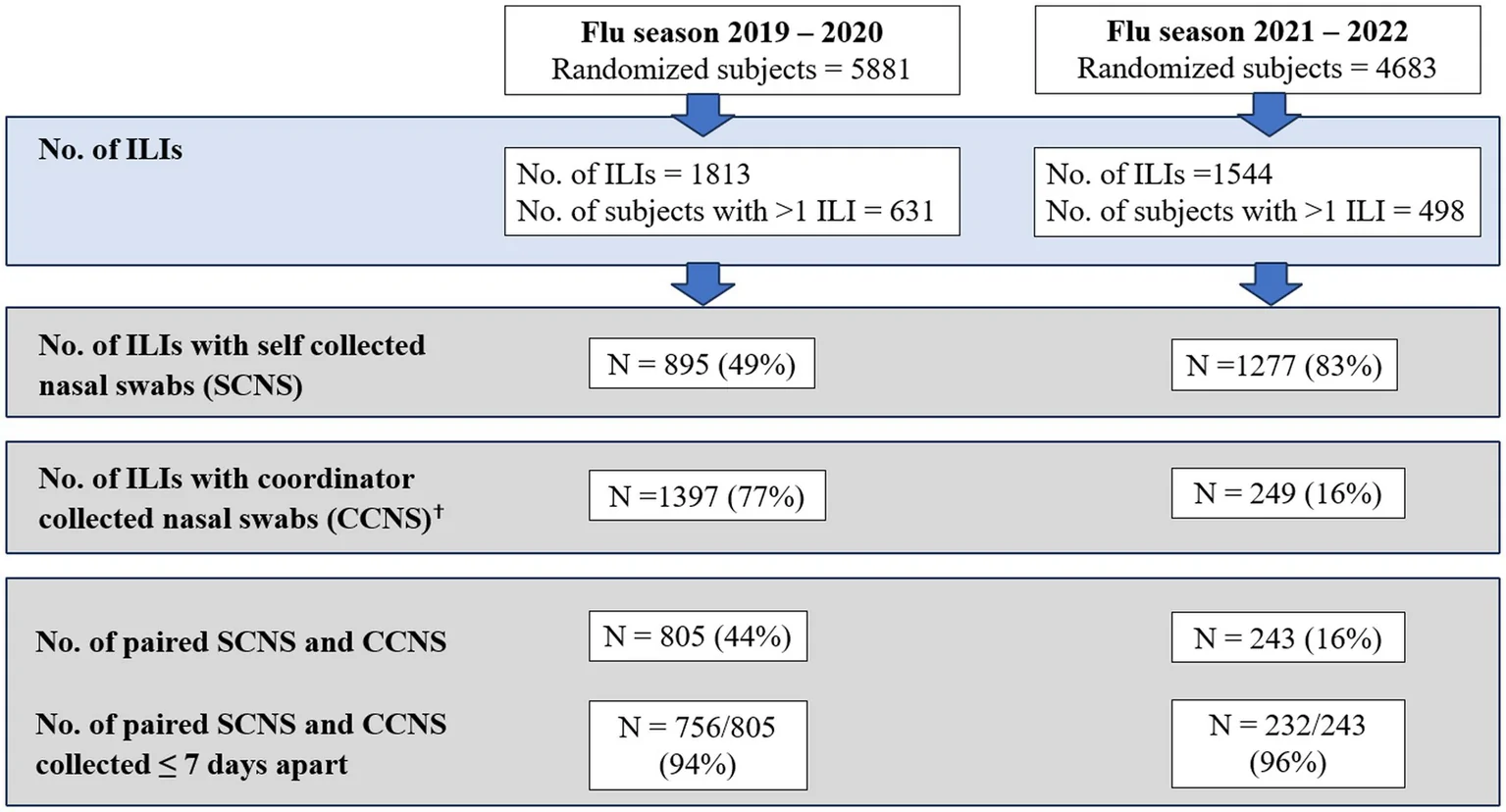
Number of self-collected nasal swabs (SCNS) and coordinator-collected nasal swabs (CCNS) during the 2019/2020 and 2021/2022 influenza season for subjects with influenza-like-illness (ILI) enrolled in the PAIVED trial. ✝More paired swabs were collected in 2019/20 (44.4%) than in 2021/22 (15.7%) due to clinic visits being made optional during the latter influenza season. CCNS: coordinator-collected nasal swab; ILI: influenza-like illness; SCNS: self-collected nasal swabs.

A total of 10,564 participants were enrolled during the influenza seasons of 2019/2020 and 2021/2022, of whom 2,756 reported at least one ILI, and 84.4% of these subjects (2,325/2,756) provided a SNCS, CCNS or both (Figure 1). Overall, at least one nasal swab was collected during 86.8% of ILIs (2,914/3,357); SCNS were collected in 64.7% of ILIs and CCNS in 49.0% of ILIs; both swabs were collected in 31.2% of ILIs (*n* = 1,048). Of the 1,048 paired-swabs, 988 were within 7 DPSO. More paired swabs were collected in 2019/2020 (44.4% of ILIs that season) than in 2021/2022 (15.7% of ILIs that season) due to in-person research visits becoming optional during the pandemic. Forty-three percent of paired swabs were provided by HCWs enrolled in the study, 68% of paired swabs were obtained from active-duty service-members and 13.2% of subjects provided > 1 swab pairs due to multiple ILIs (Supplementary Table 1). SCNS were collected closer to the onset of symptoms (median DPSO: 3.0 days [IQR 2.0–6.0 days]) than CCNS (median DPSO: 7.0 days [IQR 4.0–9.0 days]) (*p* < 0.001), and participants were more likely to report moderate or severe symptoms at time of SCNS collection (43.9% [187/426 subjects who completed FLUPRO instrument on the day of swab collection]) than at time of CCNS collection (33.3% [142/426]) (p < 0.001). There was no difference between HCWs and non-HCWs status in the timing of SCNS or CCNS collection or overall pathogen detection rate (Supplementary Table 2).

**Figure 2:**
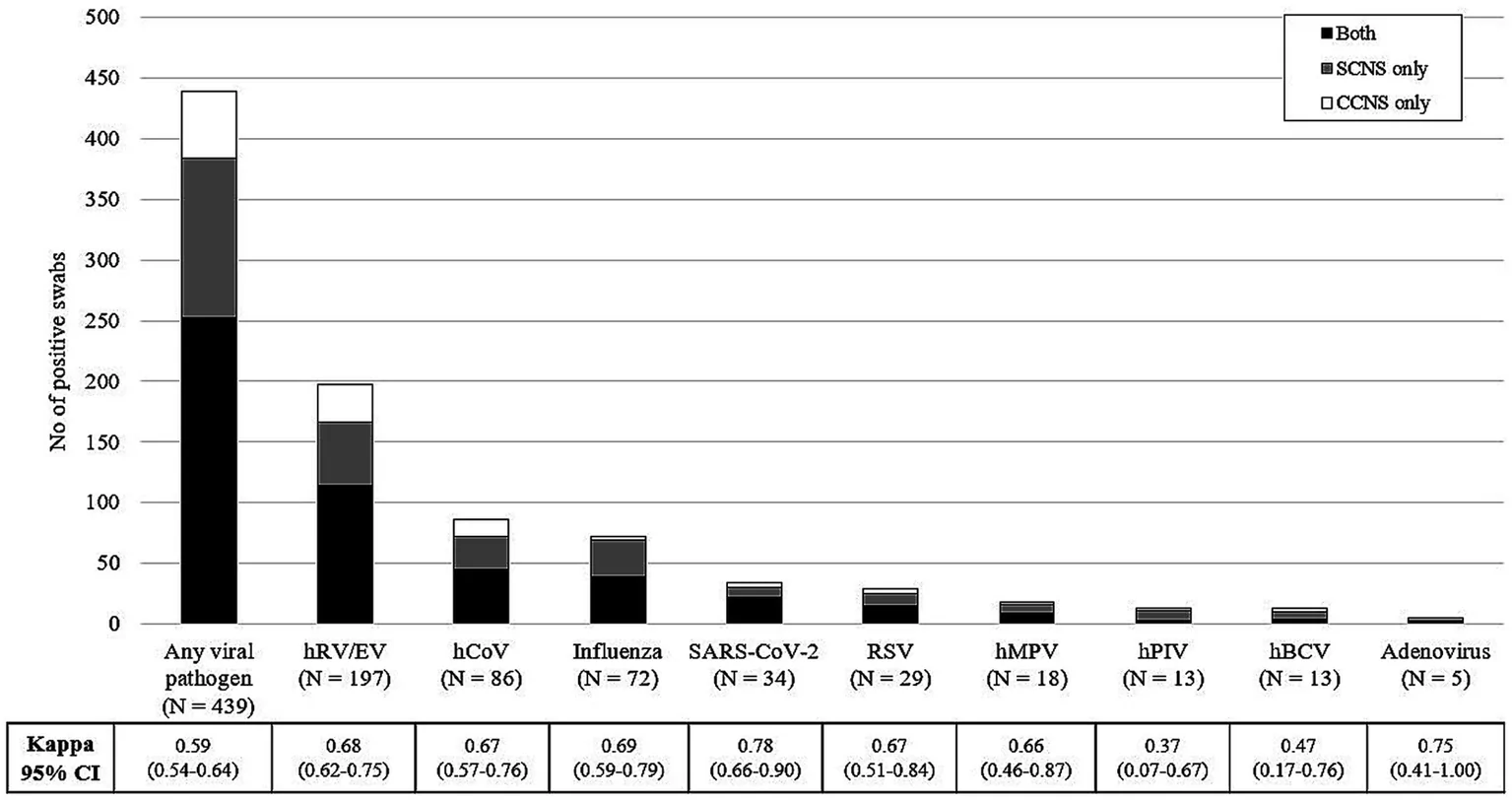
Overall distribution of respiratory viruses detected in the 988 pairs of self-collected nasal swabs (SCNS) and coordinator-collected nasal swabs (CCNS) collected ≤ 7 days apart from subjects with influenza-like-illness (ILI) enrolled in the PAIVED study. hCoV: human seasonal coronavirus (229E, OC43, NL63, or HKU1); hBCV: human bocavirus; hMPV: human metapneumovirus; influenza (A, A H1, A H3, or B); hPIV: human parainfluenza virus 1, 2, 3, or 4; RSV respiratory syncytial virus A or B; hRV/EV: human rhinovirus/enterovirus; SARS-CoV-2: Severe acute respiratory syndrome coronavirus 2. ^ǂ^CCNS: coordinator-collected nasal swab; SCNS: self-collected nasal swabs.

**Figure 3:**
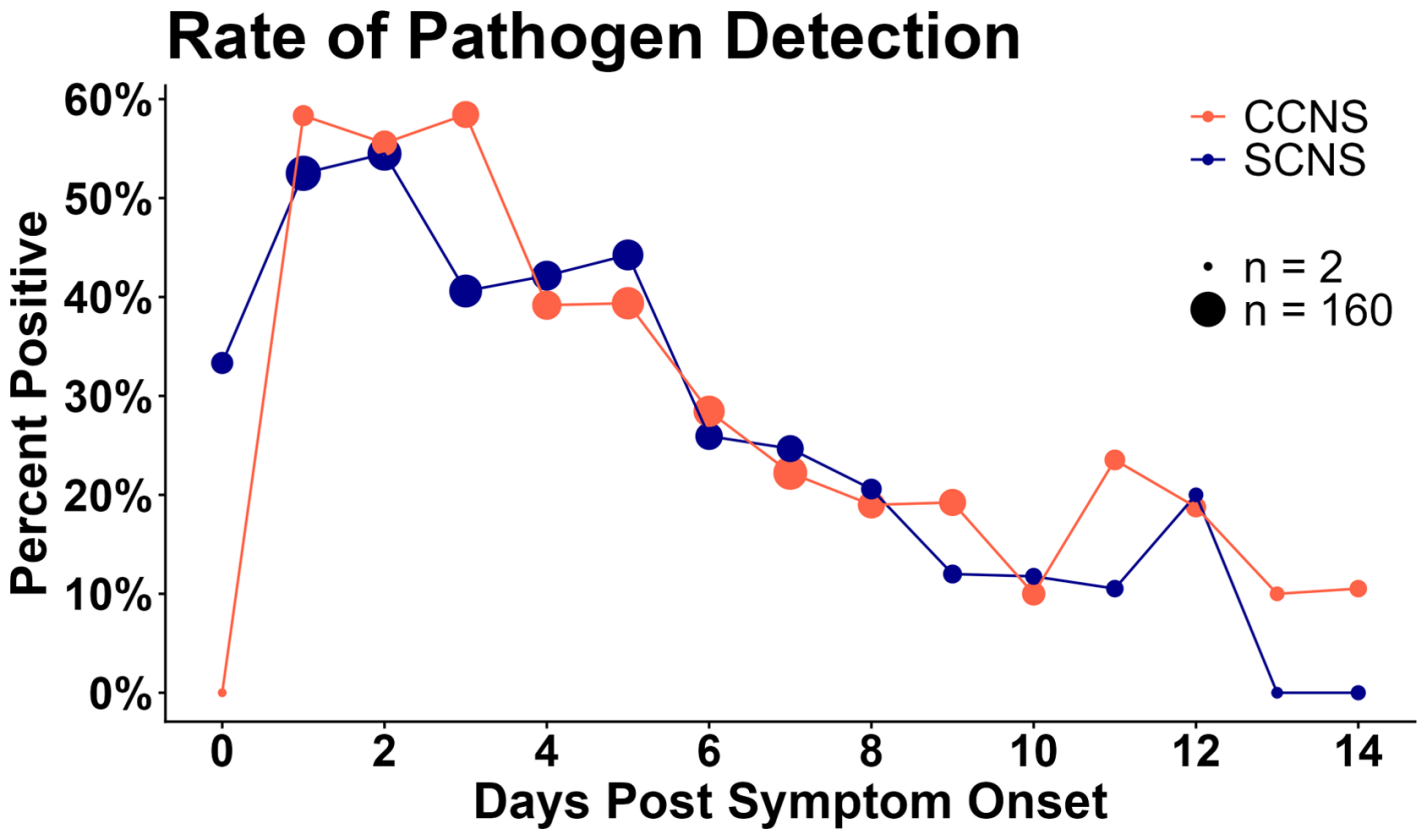
Proportion of swabs with at least one respiratory virus detected via multiplex PCR, by swab type and number of days post-symptom onset for 988 pairs of self-collected nasal swabs (SCNS) and coordinator-collected nasal swabs (CCNS). Dots are color-coded by swab type and size-coded by number of swabs. 15 SCNS and 24 CCNS were collected > 14 days after symptom onset and are not shown on the bar graph. ^ǂ^CCNS: coordinator-collected nasal swab; SCNS: self-collected nasal swabs.

We evaluated respiratory virus detection for the 988 ILIs with paired SCNS and CCNS collected within 7 days of each other. At least one respiratory virus was detected in 44.4% (*n* = 439) of ILIs using results from both swabs; considering each swab type independently, at least one respiratory virus was detected in 38.9% (*n* = 384) and 31.2% (*n* = 308) of the SCNS and CCNS, respectively (McNemar’s chi-squared = 30.242, df = 1, *p*-value < 0.001; K = 0.59). Among those pairs with at least one virus detected, most (*n* = 254) were detected on both SCNS and CCNS; an additional 148 detections were observed on SCNS only, with fewer CCNS-only detections (*n* = 65) (Figure 2). Figure 3 represents the distribution of SCNS and CCNS collected at each DPSO and the proportion of swabs with a pathogen detected. SCNS collected within 0–7 DPSO were almost three times as likely to have a pathogen detected (42.7% positive versus 14.8% among SCNS collected > 7 DPSO) and CCNS collected within 0–7 DPSO were approximately twice as likely to have a pathogen detected (38.4% positive versus 16.6% among CCNS collected > 7 DPSO). Detection rates were highest (approximately 50%) in swabs collected between 1–3 DPSO.

hRV/EV was the most common virus identified (*n* = 197), followed by seasonal coronavirus (*n* = 86) and influenza virus (*n* = 72); together, these accounted for 76% (355/467) of virus detections. There was substantial concordance between PCR detection in SCNS and CCNS for all viruses except hBCV and hPIV, which were more likely to be detected only on SCNS and were limited by the small number of positive samples (Figure 2). Substantial concordance between SCNS and CCNS was also observed for PCR detection of Influenza A (K: 0.70; SCNS+CCNS detection: 26; SCNS detection only: 19; CCNS detection only: 2) and Influenza B (K: 0.68; SCNS+CCNS detection: 13; SCNS detection only: 11; CCNS detection only: 1). A higher concordance between paired SCNS and CCNS across all pathogens was observed when both swabs were obtained within 7 DPSO versus when one or both swabs were collected > 7 DPSO (Table 2). For example, slight or no concordance between paired swabs was observed for influenza or SARS-CoV-2 when ≥ 1 swab was collected > 7 DPSO but substantial (for influenza) or almost perfect concordance (for SARS-CoV-2) was observed when both swabs were collected within 7 DPSO.

**Table 2:** Overall and pathogen-specific detection rates by detection location, with corresponding Cohen’s Kappa statistic and confidence intervals.

Pathogen detected	Detection source				Kappa, CI
	SCNS only *N* (%)	CCNS only *N* (%)	Both *N* (%)	None *N* (%)	
Both swabs collected ≤ 7 DPSO (*N* = 662)					
Any pathogen detected (*N* = 332)	78 (11.8%)	41 (6.2%)	213 (32.2%)	330 (49.8%)	0.63 (0.57, 0.69)
Rhinovirus/enterovirus (*N* = 140)	29 (4.4%)	24 (3.6%)	87 (13.1%)	522 (78.9%)	0.718 (0.65, 0.79)
Seasonal coronavirus (*N* = 71)	18 (2.7%)	13 (2%)	40 (6%)	591 (89.3%)	0.695 (0.59, 0.8)
Influenza (*N* = 58)	18 (2.7%)	2 (0.3%)	38 (5.7%)	604 (91.2%)	0.776 (0.68, 0.87)
SARS-CoV-2 (*N* = 25)	1 (0.2%)	3 (0.5%)	21 (3.2%)	637 (96.2%)	0.91 (0.82, 1)
At least 1 swab collected >7 DPSO (*N* = 326)					
Any pathogen detected (*N* = 107)	53 (16.3%)	14 (4.3%)	40 (12.3%)	219 (67.2%)	0.423 (0.31, 0.53)
Rhinovirus/enterovirus (*N* = 57)	23 (7.1%)	7 (2.1%)	27 (8.3%)	269 (82.5%)	0.592 (0.46, 0.72)
Seasonal coronavirus (*N* = 15)	9 (2.8%)	1 (0.3%)	5 (1.5%)	311 (95.4%)	0.487 (0.22, 0.76)
Influenza (*N* = 14)	12 (3.7%)	1 (0.3%)	1 (0.3%)	312 (95.7%)	0.124 (−0.11, 0.35)
SARS-CoV-2 (*N* = 9)	7 (2.1%)	1 (0.3%)	1 (0.3%)	317 (97.2%)	0.192 (−0.14, 0.52)

For 988 ILIs with paired SCNS and CCNS collected 0–7 days apart, separated by both swabs collected 0–7 DPSO and at least 1 swab collected > 7 DPSO. CCNS, coordinator-collected nasal swab; DPSO, days post-symptom onset; SCNS, self-collected nasal swabs.

Mixed infections were uncommon (*n* = 29; 1.5% of swabs) in either swab type, and there was no difference in the median number of pathogens detected by SCNS and CCNS. Among the 29 swabs with more than one pathogen detected, hRV/EV was most commonly one of the two viruses detected (62.1%) (Supplementary Figure 1).

## Discussion

We conducted a sub-analysis of a prospective clinical trial to evaluate respiratory virus detection from SCNS and CCNS in an adult population that mirrors the real-life process of patient at-home testing versus clinic-based testing. Our results support the use of SCNS for research, including research conducted during a pandemic, and for respiratory viral surveillance. Further, these findings offer important insights regarding the detection rates (which may guide trial sample size calculations and public health surveillance system design) and strategies to optimize a self-test approach. A pathogen was detected in 38.9 and 31.2% of the SCNS and CCNS, respectively. The detection rate is lower than detection rates reported in ILI surveillance studies (40–60%) (1, 18) which typically enroll participants presenting to clinics with ILIs or COVID-like-illnesses (CLI). Prior studies comparing PCR detection from SCNS and CCNS were single-site, cross-sectional studies with the performance of both swabs during an acute ILI visit (Table 1). We do acknowledge a pre-analytical limitation of variable temperature, transport and storage conditions of SCNS as collection was not uniform nor was the data systematically collected, which can affect viral detection. However, our study design replicates a real-world environment in which patients self-swab during an ILI prior to coming in for a clinic visit. We observed a lower detection rate associated with a delay between symptom onset and swab collection, with detection rates almost 50% lower in swabs collected > 7 DPSO versus those collected ≤ 7 DPSO. SCNS were more likely to be collected within 7 DPSO, resulting in higher detection rates relative to CCNS. Considering that young, healthy adults are less likely to seek care for ILI or CLI (1, 19–22), the addition of SCNS in surveillance or vaccine effectiveness studies could significantly increase the accuracy of incidence estimates for viral respiratory pathogens, especially with measures to promote swab collection within five days of symptom onset when detection rates were similar to estimates from clinic-based studies. Indeed, within the PAIVED clinical trial, SCNS led to a reduction in the delay between symptom onset and swab collection and allowed for collection of samples when in-person clinic visits could not be performed due to COVID-19 restrictions.

The concordance between SCNS and CCNS for pathogen-specific detection was similar to prior reports despite the difference in study design. For example, Thind et al. compared concordance between PCR detection using SCNS and CCNS performed during home-visits for adults and children with ILI (8). There was substantial agreement between paired swabs for influenza (K: 0.71), hRV/EV (K: 0.61) and hCoV (K: 0.77) in adults, similar to the category of agreement observed in our cohort. Agreement between paired swabs for less frequently detected viruses, such as adenovirus, hBCV, or hPIV was variable. However, it is important to note that using kappa values for lower prevalent viruses is imprecise due to small sampling and thus, pathogen-specific conclusions are limited. We observed an improvement in concordance when limiting the analysis to swabs obtained within 7 DPSO. Higher detection observed in SCNS is likely driven by earlier collection timing rather than intrinsic differences in sensitivity between self-collection and coordinator collection. This is a limitation to our study but an area for future studies specifically designed to evaluate adjusted associations under standardized timing conditions. An important consideration in our cohort was the significant proportion of HCWs (44%) who provided paired swabs and may have biased the concordance estimates. However, we did not observe a difference in the timing of swab collection or the overall pathogen detection rate in swabs obtained from HCW versus non-HCW.

The majority of our cohort consisted of younger adults including a high proportion of military servicemembers with limited comorbidities as well as a large proportion of HCWs. This may limit the generalizability of our findings to higher-risk populations, such as older adults, immunocompromised individuals, or children who are more susceptible to serious infections or co-infections as well as populations with lower health literacy and limited access to mail-based testing. Less than 2% of detections in our cohort involved more than one virus. Other limitations included the relatively small number of swabs collected early after symptom onset, especially on Day 0 (SCNS: 42; CCNS: 2) which limited an accurate estimate of the positive swabs at Day 0. Based on respiratory virus shedding kinetics, we would expect the detection proportion to be highest at Day 0. Similarly, the relatively lower number of CCNS collected within the first five DPSO (SCNS: 695 versus CCNS: 402) and the small number of detections for certain viral pathogens limited our ability to perform a comprehensive assessment of concordance. Additionally, repeated ILIs were treated as distinct clinical events, which can lead to underestimation of uncertainty. However, this was only in a minority of participants (13.2%) and our primary analyses relied on paired, within-episode comparisons between SCNS and CCNS, thus limiting the overall impact of clustering and interpretation of our findings. Nevertheless, it is an important consideration for future studies using analytic approaches that explicitly account for clustering by participants, in which they could provide more precise estimates of uncertainty.

Even so, our findings provide an important contribution to the traditional cross-sectional evaluation of concordance between PCR detection using SCNS and CCNS, underscoring that SCNS are a good alternative to health-care worker (HCW)-collected swabs for identifying ILI etiology and respiratory viral pathogen surveillance, particularly in a young, healthy population that is less likely to seek healthcare for ILIs and potentially during future pandemic research and surveillance.

## Conclusion

In conclusion, unsupervised SCNS and symptom data collection is a feasible and useful adjunct to traditional clinic-based testing (i.e., CCNS) for ILI surveillance as it enabled earlier sampling. We observed higher detection rates and better concordance between SCNS and CCNS among swabs obtained within 7 DPSO.

## Data Availability

The original contributions presented in the study are included in the article/Supplementary material, further inquiries can be directed to the corresponding author.

## PAIVED Study Group

The following people and their associated organizations served as investigators for the PAIVED trial: B. Clakley, A. E. Markelz, K. Mende, S. Merritt, T. Merritt, A. McClung, C. Murillo, D. Nall, Brooke Army Medical Center, Fort Sam Houston, TX; S. Bazan, Carl R. Darnall Army Medical Center, Fort Hood, TX; A. Swarthout-Ebarb, Lackland Air Force Base, Joint Base San Antonio, TX; D. Becher, K. Blankenship, R. Bowers, C. Coles, M. Fritschlanski, M. Grance, N. Moreno, C. Olsen, O. Ortega, H. Park, E. Parmelee, S. Pollett, S. Richard, J. Rothenberg, K. Schmidt, Henry M. Jackson Foundation, Inc. Bethesda, MD; C. Baker, J. Bowman, A. Cochran, S. Chambers, R. Colombo, B. Jones, M. Heilweil, A. King, M. Martin, C. Schofield, Madigan Army Medical Center, Joint Base Lewis-McChord, WA; S. Banks, J. McNiff, R. Smith, M. Romero, J. Dunham, T. Lalani, R. Tant, M. Cerroni, A. Austin, T. Warkentien, Naval Medical Center Portsmouth, Portsmouth, VA; C. Berjohn, K. Gyben, N. Kirkland, D. Larson, R. Maves, G. Utz, G. Zollicoffer, Naval Medical Center San Diego, San Diego, CA; C. Friend, J. Modi, A. Saperstein, D. Tilley, M. Wayman, Naval Health Clinic Annapolis, Annapolis, MD; T. Burgess, L. Kosh, R. J. O’Connell, M. Simons, A. Williams, Uniformed Services University of the Health Sciences, Bethesda, MD; A. Fries, V. Hogan, S. Purves, United States Air Force School of Aerospace Medicine, Dayton, OH; I. Barahona, A. Ganesan, J. Jefferson, L. Lawson, S. Loo, S. Loy, M. Macisaac, W. Campbell, Walter Reed National Military Medical Center, Bethesda, MD; L. Collins, L. Housel, S. Seshadri, C. Spooner, D. Hrncir, B. McClenathan, J. Brunader, C. Lohsl, E. Hall, L. Gazlay, C. Marrier, S. Bedno, A. Farmer, A. Nivens, G. Collins, M. Hussain, D. Turner, M. Richardson, J. Ritschl, A. Smith, D. Crosby, J. Simon, A. Williams, K. Matthews, C. Armstrong, D. Parish, Defense Health Agency, Immunization Healthcare Division; J. Powers III, Clinical Research Directorate, Frederick National Laboratory for Cancer Research, Frederick, MD, United States.

## Author disclaimer

The views expressed are those of the authors and do not reflect the official policy of the Uniformed Services University of the Health Sciences (USUHS); the Department of War (DoW); Department of the Army, Department of the Navy, the Department of the Air Force, the Department of War, the U. S. Government, U. S. Army Medical Dept, US Army Office of the SG, BAMC, MAMC, WRNMMC, USUHS, DHA, and the Henry M. Jackson Foundation for the Advancement of Military Medicine, Inc. (HJF). Mention of trade names, commercial products, or organizations does not imply endorsement by the U. S. Government. Investigators followed human subjects protection 45CFR46 policies. Some authors (MK, CS, JM, DL, AF, TB) are military service members and employees of the U.S. Government. This work was prepared as part of their official duties. Title 17 U.S.C. 105 provides that “Copyright protection under this title is not available for any work of the United States Government.” Title 17 U.S.C. 101 defines a United States Government work as a work prepared by a military service member or employee of the United States Government as part of that person’s official duties.
