# Supplementary material for: Participant self-collected versus research coordinator-collected nasal swabs for respiratory viral surveillance following influenza-like illness
Source: Front Public Health. 2026 Aug 19;14:1846269. doi: 10.3389/fpubh.2026.1846269 (PMC13533934; doi:10.3389/fpubh.2026.1846269)
Supplement: Supplementary file 1 [file Supplementary_file_1.docx]

**SUPPLEMENTARY TABLE 1.** Demographic characteristics of subjects with ILI(s) who provided paired self-collected and coordinator collected nasal swabs vs. those who did not provide paired swabs.

|  | **Swab pair 0-7 days apart** | |  |
| --- | --- | --- | --- |
|  | **Yes**  N = 988^1^ | **No**  N = 60^1^ | **p-value**^2^ |
| **First swab DPSO** | 4.0 (2.0, 6.0) | 2.0 (1.0, 3.5) | 0.000 |
| **Second swab DPSO** | 6.0 (4.0, 8.5) | 13.0 (10.0, 14.0) | 0.000 |
| **Any virus detected** | 439 (44.4%) | 30 (50.0%) | 0.400 |
| **Season** |  |  | 0.359 |
| 2019/20 | 756 (76.5%) | 49 (81.7%) |  |
| 2021/22 | 232 (23.5%) | 11 (18.3%) |  |
| **Age at enrollment** | 35.5 (29.0, 45.1) | 36.9 (29.9, 48.6) | 0.129 |
| **Sex** |  |  | 0.232 |
| Male | 531 (53.7%) | 37 (61.7%) |  |
| Female | 457 (46.3%) | 23 (38.3%) |  |
| **Race/Ethnicity** |  |  | 0.119 |
| White | 522 (52.8%) | 40 (66.7%) |  |
| Hispanic or Latino | 189 (19.1%) | 5 (8.3%) |  |
| Black or African American | 140 (14.2%) | 7 (11.7%) |  |
| Other/Unknown | 137 (13.9%) | 8 (13.3%) |  |
| **Healthcare worker status** |  |  | 0.753 |
| No | 558 (56.5%) | 32 (53.3%) |  |

| Yes | 426 (43.1%) | 28 (46.7%) |  |
| --- | --- | --- | --- |
| Unknown/Not documented | 4 (0.4%) | 0 (0.0%) |  |
| **Military status** |  |  | 0.946 |
| Active duty | 671 (67.9%) | 41 (68.3%) |  |
| Non-active duty (retired/dependent) | 317 (32.1%) | 19 (31.7%) |  |
| ^1^Median (Q1, Q3); n (%) | | | |
| ^2^Wilcoxon rank sum test; Pearson's Chi-squared test; Fisher's exact test | | | |

**SUPPLEMENTARY TABLE 2.** Comparison of participants who were health-care workers vs. non-health-care workers with regards to timing of swab collection from symptom onset and overall viral pathogen detection by PCR.

|  | **Non-HCW (n=590)** | **HCW (n=454)** | **p-value** |
| --- | --- | --- | --- |
| Paired swabs collected 0-7 days apart | 558 (94.6%) | 426 (93.8%) | 0.6 |
| Day from symptom onset to SCNS (median, Q1-Q3) | 4.0 (2.0, 6.0) | 3.0 (2.0, 6.0) | 0.3 |
| Days from symptom onset to CCNS (median, Q1-Q3) | 7.0 (5.0, 9.0) | 6.0 (4.0, 9.0) | 0.2 |
| Any pathogen detected on SCNS | 234 (39.7%) | 178 (39.2%) | 0.9 |
| Any pathogen detected on CCNS | 117 (30.0%) | 136 (30.0%) | > 0.9 |
| Paired swabs – both positive or both negative | 477 (80.8%) | 356 (78.4%) | 0.3 |

Abbreviations: CCNS: coordinator-collected nasal swab; HCW: health-care worker; SCNS: self-collected nasal swabs

**SUPPLEMENTARY FIGURE 1.** UpSet plot showing coinfections detected on 1,976 paired swabs collected 0-7 days apart. Bar chart (top) shows count of each coinfection group detected while intersection plot (bottom) portrays the viruses comprising each coinfection group.
